# Association of Perioperative Plasma Neutrophil Gelatinase-Associated Lipocalin Levels with 3-Year Mortality after Cardiac Surgery: A Prospective Observational Cohort Study

**DOI:** 10.1371/journal.pone.0129619

**Published:** 2015-06-08

**Authors:** Dennis G. Moledina, Chirag R. Parikh, Amit X. Garg, Heather Thiessen-Philbrook, Jay L. Koyner, Uptal D. Patel, Prasad Devarajan, Michael G. Shlipak, Steven G. Coca, TRIBE-AKI Consortium

**Affiliations:** 1 Section of Nephrology, Department of Medicine, Yale University School of Medicine, New Haven, Connecticut, United States of America; 2 VA CT Healthcare System, West Haven, Connecticut, United States of America; 3 Program of Applied Translational Research, Department of Medicine, Yale University School of Medicine, New Haven, Connecticut, United States of America; 4 Division of Nephrology, Department of Medicine, Western University, London, Ontario, Canada; 5 Institute for Clinical Evaluative Sciences, Toronto, Ontario, Canada; 6 Section of Nephrology, Department of Medicine, University of Chicago, Chicago, Illinois, United States of America; 7 Duke Clinical Research Institute, Duke University School of Medicine, Durham, North Carolina, United States of America; 8 Division of Nephrology and Hypertension, Cincinnati Children’s Hospital Medical Center, Cincinnati, Ohio, United States of America; 9 Division of General Internal Medicine, San Francisco VA Medical Center, University of California, San Francisco, California, United States of America; University of Sao Paulo Medical School, BRAZIL

## Abstract

**Background:**

Higher levels of plasma neutrophil gelatinase-associated lipocalin (pNGAL) are an early marker of acute kidney injury and are associated with increased risk of short-term adverse outcomes. The independent association between pNGAL and long-term mortality is unknown.

**Methods:**

In this prospective observational cohort study, we studied 1191 adults who underwent cardiac surgery between 2007 and 2009 at 6 centers in the TRIBE-AKI cohort. We measured the pNGAL on the pre-operative and first 3 post-operative days and assessed the relationship of peri-operative pNGAL concentrations with all-cause mortality.

**Results:**

During a median follow-up of 3.0 years, 139 participants died (50/1000 person-years). Pre-operative levels of pNGAL were associated with 3-year mortality (unadjusted HR 1.96, 95% CI 1.34,2.85) and the association persisted after adjustment for pre-operative variables including estimated glomerular filtration rate (adjusted HR 1.48, 95% CI 1.04–2.12). After adjustment for pre- and intra-operative variables, including pre-operative NGAL levels, the highest tertiles of first post-operative and peak post-operative pNGAL were also independently associated with 3-year mortality risk (adjusted HR 1.31, 95% CI 1.0–1.7 and adjusted HR 1.78, 95% CI 1.2–2.7, respectively). However, after adjustment for peri-operative changes in serum creatinine, there was no longer an independent association between the first post-operative and peak post-operative pNGAL and long-term mortality (adjusted HR 0.98,95% CI 0.79–1.2 for first pNGAL and adjusted HR 1.19, 95% CI 0.87–1.61 for peak pNGAL).

**Conclusions:**

Pre-operative pNGAL levels were independently associated with 3-year mortality after cardiac surgery. While post-operative pNGAL levels were also associated with 3-year mortality, this relationship was not independent of changes in serum creatinine. These findings suggest that while pre-operative pNGAL adds prognostic value for mortality beyond routinely available serum creatinine, post-operative pNGAL measurements may not be as useful for this purpose.

## Introduction

Neutrophil gelatinase-associated lipocalin (NGAL) is a member of the lipocalin family that has gained prominence after the discovery that NGAL is markedly upregulated when kidney tubular cells are damaged.[1] Increases in the concentration of plasma NGAL (pNGAL) appear to be an important early biomarker of acute kidney injury (AKI) across various ischemic, septic, or nephrotoxic insults to the kidney. Plasma NGAL is elevated as early as 2 hours after cardiac surgery and shows a high degree of sensitivity and specificity for development of clinical AKI (diagnosed by rise in creatinine a few days later).[2–4] Plasma NGAL also adds to AKI risk prediction when added to traditional models.[5–8]

We have shown that an early increase in pNGAL after adult cardiac surgery was associated with short-term outcomes including a higher risk of AKI as defined by serum creatinine elevations, prolonged hospitalization, receipt of acute dialysis and death during hospitalization.[5,8] We demonstrated that post-operative rise in urinary NGAL in the setting of AKI in cardiac surgery is associated with long-term outcomes (3-year mortality).[6] However, the association of post-operative pNGAL with long-term mortality is currently unknown. As established with other biomarkers, such as troponin,[9,10] the clinical utility of elevated pNGAL would also improve if it independently associates with longer-term outcomes. However, it remains possible that acute increases in pNGAL do not provide any addition prognostic information beyond acute increases in serum creatinine, as pNGAL can rise due to reduced urinary excretion from reductions in glomerular filtration rate.[11] In fact, we previously demonstrated that the adjusted odds ratio of the upper quintile of pNGAL for the development of AKI was attenuated from approximately 5 down to approximately 2 when the first post-operative serum creatinine value was added to the multivariable model.[5] These observations provided the impetus to examine the independent association of peri-operative pNGAL levels with 3-year mortality in the Translational Research Investigating Biomarker Endpoints in AKI (TRIBE-AKI) cohort.

## Patients And Methods

### Study Population

The Translational Research Investigating Biomarker Endpoints for Acute Kidney Injury (TRIBE-AKI) study is a prospective cohort of 1219 adults at moderate to high risk of AKI who underwent cardiac surgery (CABG or valve surgery).[5] Participants were enrolled at six academic medical centers in North America from July 2007 through December 2009. Full study details have been previously described.[5] In brief, we collected plasma specimens preoperatively and daily for up to 5 days after surgery. We stopped specimen collection on post-operative day 3 in patients who were transferred to a hospital ward from the intensive care unit without evidence of an increase in serum creatinine. Patients who died during the index hospitalization for surgery (n = 20) or did not have plasma NGAL levels measured (n = 8) were excluded from this analysis leaving 1191 patients included in this analysis. Written informed consent was obtained from all patients or their proxy decision-makers. The TRIBE-AKI study was approved by the respective institutional review boards at Yale University-School of Medicine/Yale-New Haven Hospital (New Haven, CT, USA), University of Colorado Health Sciences Center (Denver, CO, USA), Western University/London Health Sciences Center (London, ON, Canada), University of Chicago-School of Medicine, Danbury Hospital (Danbury, CT, USA), and Duke University Medical Center (Durham, NC, USA).

### Measurement of Plasma NGAL

We evaluated 3 pNGAL values: 1) pre-operatively, 2) first post-operative (0–6 hours after surgery), and 3) peak post-operative in days 1–3 following surgery. We measured pNGAL using the Triage NGAL immunoassay in conjunction with the Triage Meter (Biosite, Inc., San Diego, CA) in two batches 7 months apart. The Triage assay has a detection range of 60 to 1300 ng/ml with a CV of 10% to 15%,[5,8] and all biomarker measurements were completed before ascertainment of vital status. The personnel measuring the biomarkers were blinded to clinical outcomes, including AKI and vital status.

### Measurement of Covariates

We recorded serum creatinine values obtained in routine clinical care for every patient throughout the hospital stay. All pre-operative creatinine values were measured within two months prior to surgery. The pre- and post-operative serum creatinine levels were performed in the same clinical laboratory for each patient at all sites. We estimated preoperative GFR (eGFR) using the Chronic Kidney Disease Epidemiology Collaboration equation.[12] We defined AKI clinically by a change in serum creatinine of ≥ 50% or ≥ 0.3 mg/dL from pre- to the peak post-operative value.[13] We collected preoperative characteristics, operative details, and post-operative complications using definitions of the Society of Thoracic Surgeons.[5]

### Mortality

We obtained vital status after discharge through various mechanisms (and cross-referenced when possible). For those living in the United States, we performed phone calls to patients’ homes, searched the National Death Index, and reviewed hospital records. For Canadian participants (those enrolled into the TRIBE-AKI study in London, Ontario), we performed phone calls and used data held at the Institute for Clinical Evaluative Sciences to acquire vital status. These datasets were linked using unique encoded identifiers and analyzed at the Institute for Clinical Evaluative Sciences (ICES). The death status and date of death were recorded through the last follow-up date of February 21, 2012. There was 100% ascertainment of vital status on the cohort.

### Statistical Analysis

We used Cox proportional hazards regression with robust sandwich variance estimators (accounting for clustering within centers) to examine the association between plasma NGAL and time to death from the date of surgery. Biomarker concentrations were expressed as tertiles with the lowest tertile as the reference group. In model 1, we adjusted for several pre-operative and intraoperative variables including the following: age (per year), sex, race (white vs. non-white), non-elective surgery, diabetes, hypertension, congestive heart failure (CHF), myocardial infarction (MI), type of surgery (coronary artery bypass graft alone or valve repair alone vs. both), pre-op eGFR (>60 vs. ≤60 ml/min/1.73 m^2^), pre-op urine albumin to creatinine ratio, cardiopulmonary bypass time (>120 minutes vs. ≤120 minutes or no CPB time), and clinical center. Model 2 applied only to the first post-operative and peak post-op NGAL levels, since it included all variables from model 1 and further adjusted for the pre-operative NGAL level. For model 3, we included all variables from model 1 and further adjusted for the change in serum creatinine value (corresponding to the same time point value of NGAL) from the pre-operative baseline. We used Schoenfeld residuals to confirm the proportional-hazards assumption. Small cell counts are only presented for data collected by TRIBE-AKI and not from ICES data holdings. All analyses were performed is SAS version 9.3 (SAS institute, Cary, NC) and R 2.15.0 (R Foundation for Statistical Computing, Vienna, Austria).

## Results

The baseline characteristics of patients by peak post-operative pNGAL are presented in **Table 1** and baseline characteristics of patients by pre-operative pNGAL are presented in **S1 Table.** During a median follow-up of 3 years (interquartile range 2.2–3.6), 139 participants died overall (50 deaths per 1000 person-years). At the time of the surgery, patients had a mean age of 71 years, 68% were men, and 94% were of white race. As compared to the lowest pre-operative pNGAL, patients in the highest tertile had lower eGFR, more likely to have valve surgery, and had longer hospital and intensive care unit stay. As compared to patients with lowest peak plasma NGAL, patients in the highest tertile had lower eGFR, more likely to have valve surgery, and more likely to have a higher cross clamp time.

**Table 1:** Baseline Characteristics by Peak Post-operative Plasma NGAL Tertiles.

Characteristic	Overall (n = 1191)	Peak Post-op Plasma NGAL Tertiles			
		Tertile 1: < 155 (n = 403, 33.8%)	T2: 155–251 (n = 401, 33.7%)	T3: >251 (n = 387, 32.5%)	P value
**Demographics**					
Age at the time of surgery, mean (SD)	71.36 (10.08)	70.59 (10.41)	72.03 (9.91)	71.47 (9.86)	0.16
Men	813 (68%)	275 (68%)	267 (67%)	271 (70%)	0.58
White Race	1114 (94%)	374 (93%)	376 (94%)	364 (94%)	0.75
**Medical History (time of surgery)**					
Diabetes	473 (40%)	171 (42%)	152 (38%)	150 (39%)	0.38
Hypertension	942 (79%)	321 (80%)	308 (77%)	313 (81%)	0.35
LVEF <40%	118 (10%)	34 (8%)	47 (12%)	37 (10%)	0.29
Previous myocardial infarction	306 (26%)	106 (26%)	102 (25%)	98 (25%)	0.94
eGFR (mL/min per 1.73 m^2^), mean (SD)	67.54 (19.3)	72.28 (17.31)	68.38 (18.7)	61.72	<.001
Pre-op eGFR stage 1	144 (12%)	65 (16%)	48 (12%)	31 (8%)	<.001
Pre-op eGFR stage 2	640 (54%)	244 (61%)	220 (55%)	176 (45%)	
Pre-op eGFR stage 3	372 (31%)	89 (22%)	125 (31%)	158 (41%)	
Pre-op eGFR stage 4	35 (3%)	5 (1%)	8 (2%)	22 (6%)	
Serum Creatinine (mg/DL), median [IQR[	1.0 [0.9, 1.2]	1 [0.8, 1.1]	1 [0.9, 1.2]	1.1 [0.9, 1.4]	<.001
Urine albumin to creatinine					
< = 10.0	458 (38%)	193 (48%)	131 (33%)	134 (35%)	0.001
10.0–30	344 (29%)	98 (24%)	133 (33%)	113 (29%)	
30–300	320 (27%)	95 (24%)	116 (29%)	109 (28%)	
> = 300	69 (6%)	17 (4%)	21 (5%)	31 (8%)	
**Surgical Characteristics**					
Elective Surgery	946 (79%)	309 (77%)	335 (84%)	302 (78%)	0.04
Surgery,					
CABG	579 (49%)	262 (65%)	175 (44%)	142 (37%)	<.001
CABG + Valve	267 (22%)	63 (16%)	95 (24%)	109 (28%)	
Valve	345 (29%)	78 (19%)	131 (33%)	136 (35%)	
Off-pump	124 (10%)	82 (20%)	27 (7%)	15 (4%)	<.001
Re-do surgery	19 (2%)	5 (1%)	3 (1%)	11 (3%)	0.04
Perfusion time (minutes), mean (SD)	113.44 (59.66)	91.78 (57.98)	114.53 (53.43)	133.76 (60.16)	<.001
Cross-clamp time (minutes), mean (SD)	77.4 (44.48)	63.07 (42.86)	76.81 (38.56)	92.28 (46.98)	<.001
**Post-operative Complications**					
Clinical AKI	49 (4%)	5 (1%)	12 (3%)	32 (8%)	<.001
Oliguria in first day	15 (1%)	6 (2%)	2 (1%)	7 (2%)	0.22
Delta Peak Serum Creatinine (mg/dL), mean (SD)	0.1 [0, 0.3]	0.1 [-0.03, 0.2]	0.1 [0, 0.27]	0.21 [0.08, 0.5]	<.001
ICU LOS, median [IQR]	2 [1, 3]	2 [1, 2]	2 [1, 3]	2 [1, 4]	<.001
Hospital LOS, median [IQR]	6 [5, 8]	6 [5, 7]	6 [5, 8]	7 [6, 10]	<.001

Abbreviations and definitions:

Data presented as number (percent) unless otherwise specified.

LVEF- left ventricular ejection fraction, eGFR- estimate glomerular filtration rate, ICU- intensive care unit, LOS- length of stay, IQR- interquartile range; Clinical AKI defined as change in serum creatinine of ≥ 100% from pre- to the peak post-operative value. Oliguria defined as a patient who had <125 cc in 6 hours or <500 cc urine output in 24 hours

Note: To convert serum creatinine values to mmol/L, multiply by 88.4.

Small cell counts are only presented for data collected by TRIBE-AKI and not from ICES data holdings.

### Association of Pre-op Plasma NGAL and Serum Creatinine with 3-year Mortality

The distribution of pre-op pNGAL levels was generally lower than the post-op levels. 589 of 1191 (49%) had pNGAL levels below the lower limit of detection (< 60 pg/ml). Patients with pre-op pNGAL levels in the 3^rd^ tertile (> 77 pg/ml) had nearly 2-fold increased risk of death compared to patients with plasma NGAL that was below the lower limit of detection. After adjustment for pre-op characteristics and pre-op eGFR, the 2^nd^ and 3^rd^ tertile of pNGAL were still independently associated with long-term mortality (**model 2, Table 2;** adjusted HR 1.38 [95% CI 1.11, 1.72] and 1.48 [95% CI 1.04–2.12], respectively). We found similar association between pre-operative pNGAL values and long-term mortality when patients who experienced in-hospital mortality (n = 20) were included in this analysis. In addition, the results were unchanged when the analysis was adjusted for the presence of preoperative CKD (eGFR <60 mL/min/1.73m^2^, or albumin to creatinine ratio >30), rather than preoperative eGFR as a continuous variable (data not shown). Patients with pre-op serum creatinine concentrations in the 3^rd^ tertile (> 1.1 mg/dL) were also at increased risk for death, although adjustment for covariates attenuated the signal (**model 1, Table 2;** adjusted HR 1.47, 95% CI 0.92–2.33).

**Table 2:** Risk of Death by Pre-operative Biomarkers.

Tertile	Biomarker Range	Death Rate†	HR (95% CI)		
			Unadjusted	Model 1§	Model 2‡
**Plasma NGAL Tertiles (ng/ml)** *					
T1	<60¶	37.1	1.0 (referent)	1.0 (referent)	1.0 (referent)
T2	60–77	49.4	1.35 (1.1,1.65)	1.39 (1.13,1.71)	1.38 (1.11,1.72)
T3	>77	72.7	1.96 (1.34,2.85)	1.51 (1.03,2.23)	1.48 (1.04,2.12)
**Serum Creatinine Tertiles (mg/dl)**					
T1	<0.9	42.0	1.0 (referent)	1.0 (referent)	N/A
T2	0.9–1.1	41.3	0.99 (0.8,1.22)	1.01 (0.81,1.25)	N/A
T3	>1.1	70.3	1.67 (1.2,2.32)	1.47 (0.92,2.33)	N/A

†Mortality Rate per 1000 patient-years adjusted for site

¶60 ng/ml was the lower limit of detection for the assay

§Model 1: Adjusted for Age (per year), sex, white race, non-elective surgery, diabetes, hypertension, centre, Congestive heart failure (CHF), Myocardial Infarction (MI), Pre-op urine albumin to creatinine ratio, and Type of surgery (CABG or valve vs. all others).

‡Model 2: Model 1 + pre-op eGFR

*: Tertiles of Pre-operative Plasma NGAL were unequally distributed with 52.5% of patients had pNGAL below the lower limit of detection (T1 = 589; T2 = 156; T3 = 376).

### Association of First postoperative plasma NGAL and Serum Creatinine with 3-year Mortality

**Fig 1:**
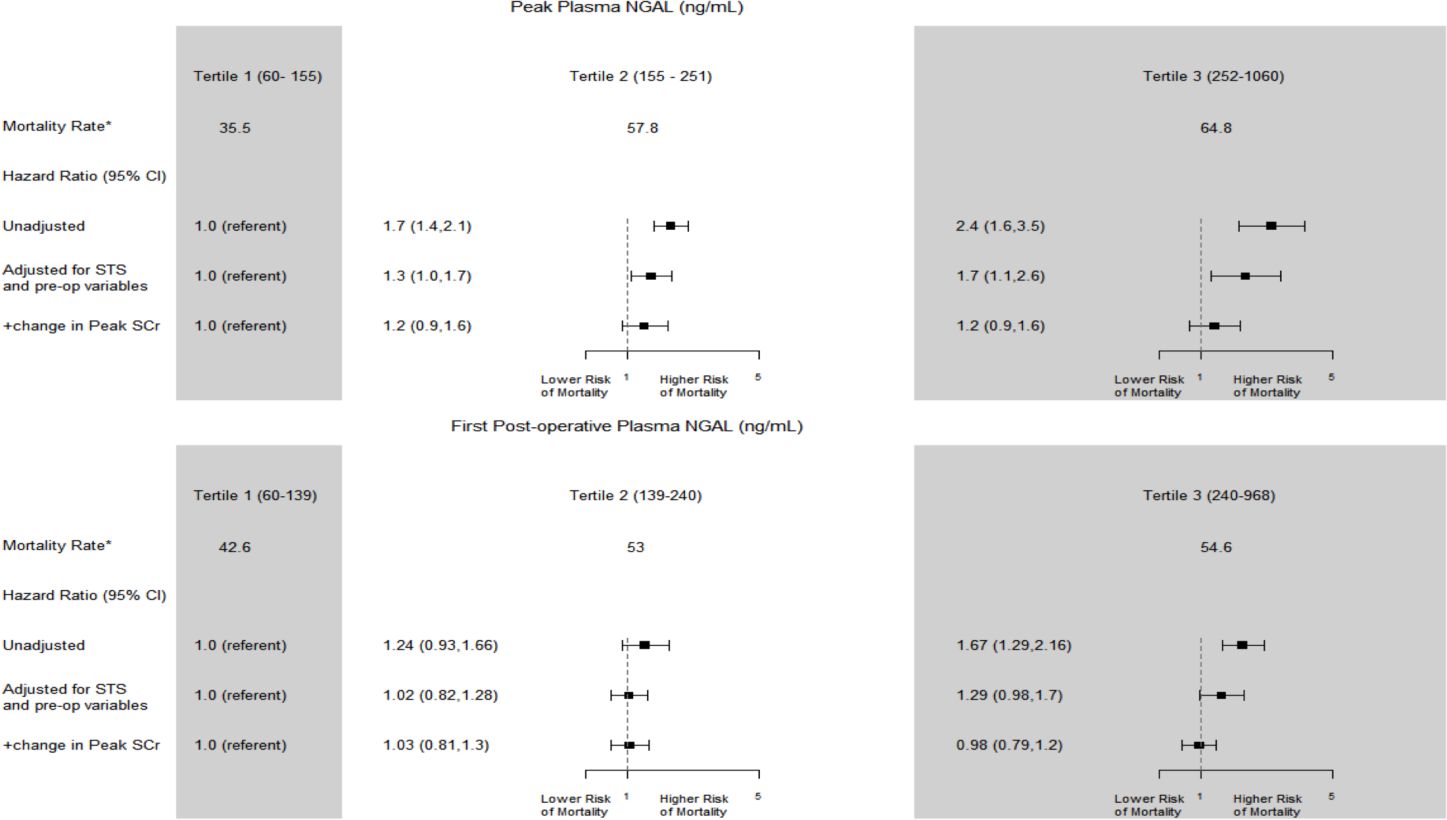
Biomarker levels and the risk of death. *Mortality Rate per 1000 patient-years adjusted for site.

The distribution of pNGAL concentrations were markedly greater for the first post-operative values (median 183, IQR 118–266) compared to the pre-operative values. Unadjusted mortality rates rose from 42.6 to 53.0 and 54.6 for the first, second and third tertiles, respectively. After adjustment for pre-operative characteristics and pre-operative pNGAL, the risk attenuated but patients in the 3^rd^ tertile (vs. the first tertile) were still at increased risk (**model 2, Table 3**; adjusted HR 1.31, 95% CI 1.03,1.67). There was no longer an independent association between the first post-operative pNGAL when adjusted for change in first creatinine from preoperative value (**model 3, Table 3,** and **Fig 1**; adjusted HR 0.98, 95% CI 0.79–1.2). The 3^rd^ tertile of the first post-operative serum creatinine was also independently associated with long-term mortality (**model 2, Table 3;** adjusted HR 2.11, 95% CI 1.78,2.5).

**Table 3:** Risk of Death by First Post-operative Biomarkers.

Tertile	Biomarker Range	Death Rate†	HR (95% CI)			
			Unadjusted	Model 1§	Model 2‡	Model 3†
**Plasma NGAL Tertiles (ng/ml)**						
T1	< 139	42.6	1.0 (referent)	1.0 (referent)	1.0 (referent)	1.0 (referent)
T2	139–240	53.0	1.24 (0.93,1.66)	1.02 (0.82,1.28)	1.01 (0.82,1.25)	1.03 (0.81,1.3)
T3	>240	54.6	1.67 (1.29,2.16)	1.29 (0.98,1.7)	1.31 (1.03,1.67)	0.98 (0.79,1.2)
**Serum Creatinine Tertiles (mg/dl)**						
T1	<0.9	37.9	1.0 (referent)	1.0 (referent)	1.0 (referent)	NA
T2	0.9–1.19	51.9	1.5 (1.11,2.02)	1.57 (1.21,2.05)	1.52 (1.17,1.97)	NA
T3	>1.19	70.9	2.32 (1.83,2.93)	2.31 (1.84,2.91)	2.11 (1.78,2.5)	NA

†Mortality Rate per 1000 patient-years adjusted for site

§ Model 1: Adjusted for Age (per year), sex, white race, CPB time > 120 minutes, non-elective surgery, pre-op eGFR, diabetes, hypertension, centre, Congestive heart failure (CHF), Myocardial Infarction (MI), Pre-op urine albumin to creatinine ratio, and Type of surgery (CABG or valve vs. all others).

‡Model 2: Model 1 + pre-op biomarker

†Model 3: Model 1 + change in serum creatinine value from pre-op to first post-op

### Association of Peak postoperative plasma NGAL and Serum Creatinine with 3-year Mortality

The distribution of peak post-operative pNGAL was median 201, IQR 134–281. The unadjusted mortality rates were 35.5, 57.8 and 64.8 per 1000 person years in the first, second and third tertiles of peak post-operative pNGAL levels. After adjustment for pre-operative characteristics and pre-op pNGAL, the third tertile of peak pNGAL was still independently associated with long-term mortality when compared to the lowest tertile (**model 2, Table 4** and **Fig 1;** adjusted HR 1.78, 95% CI 1.17–2.69). However, as seen with other analyses above, this association between pNGAL and long-term mortality was attenuated and became non-significant after adjustment for change in serum creatinine (**model 3, Table 4** and **Fig 1**; adjusted HR 1.2, 95% CI 0.9–1.6). The third tertile of peak post-op serum creatinine concentration was also strongly associated with long-term mortality (**model 2, Table 4;** adjusted HR 3.1, 95% CI 2.5–3.8). Changes in pNGAL values from baseline to first postoperative or from baseline to peak postoperative time point were not associated with 3-year mortality (data not shown).

**Table 4:** Risk of Death by Peak Biomarkers.

Tertile	Biomarker Range	Death Rate†	HR (95% CI)			
			Unadjusted	Model 1§	Model 2‡	Model 3†
**Plasma NGAL Tertiles (ng/ml)**						
T1	< 155	35.5	1.0 (referent)	1.0 (referent)	1.0 (referent)	1.0 (referent)
T2	155–251	57.8	1.7 (1.37,2.11)	1.34 (1.04,1.71)	1.37 (1.02,1.83)	1.23 (0.93,1.63)
T3	>251	64.8	2.38 (1.6,3.53)	1.73 (1.13,2.63)	1.78 (1.17,2.69)	1.19 (0.87,1.61)
**Serum Creatinine Tertiles (mg/dl)**						
T1	<1.01	36.1	1.0 (referent)	1.0 (referent)	1.0 (referent)	NA
T2	1.01–1.31	47.0	1.33 (0.87,2.04)	1.45 (0.93,2.25)	1.43 (0.92,2.21)	NA
T3	>1.31	80.4	2.82 (2.13,3.74)	3.1 (2.53,3.8)	2.99 (2.44,3.65)	NA

†Mortality Rate per 1000 patient-years adjusted for site

§Model 1: Adjusted for Age (per year), sex, white race, CPB time > 120 minutes, non-elective surgery, pre-op eGFR, diabetes, hypertension, center, Congestive heart failure (CHF), Myocardial Infarction (MI), Pre-op urine albumin to creatinine ratio, and Type of surgery (CABG or valve vs. all others).

‡Model 2: Model 1 + pre-op biomarker

†Model 3: Model 1 + change in serum creatinine value from pre-op to peak

#### Adjusted for STS variables and pre-op variables

Adjusted for Age (per year), sex, white race, CPB time > 120 minutes, non-elective surgery, pre-op eGFR, diabetes, hypertension, centre, Congestive heart failure (CHF), Myocardial Infarction (MI), Pre-op urine albumin to creatinine ratio, and Type of surgery (CABG or valve vs. all others)

#### +change in Peark SCr

**Model Adjusted for STS variables and pre-op variables** + change in peak serum creatinine from pre-op

### Correlation between peri-operative plasma NGAL and other variables

The correlation between perioperative pNGAL and relevant perioperative variables is presented in **S2 Table.** There was modest positive correlation of pre-operative pNGAL with first and peak post-operative pNGAL values (R^2^: 0.33 and 0.34, respectively). The first and peak post-operative pNGAL values also had a modest correlation with changes in serum creatinine from baseline to first post-operative (R^2^: 0.24) and from baseline to peak post-operative (R^2^: 0.28), respectively. Additionally, at each time point, pNGAL values were positively correlated with corresponding absolute serum creatinine values (R^2^: Pre-op: 0.30, First Post-op 0.35 and Peak post-op 0.38).

### Analysis by AKI status

In our previous study, urine NGAL significantly associated with mortality only in individuals with clinical AKI.[7] Similarly, we conducted additional analysis stratified by clinical AKI status to test the association of post-operative NGAL values with long-term mortality (**S3 Table**) but we did not find robust interaction by AKI status.

## Discussion

Plasma NGAL has been previous demonstrated to be independently associated with short-term outcomes, and has the potential to provide incremental information regarding long-term risk. There have been a number of clinical studies evaluating pNGAL as an early and prognostic marker in AKI;[1,14] however, data for long-term outcomes is scarce. In this study we found that pNGAL levels were higher pre- and post-operatively in patients that were less likely to survive long-term, yet the associations for post-operative NGAL were markedly attenuated and became non-significant after accounting for changes in serum creatinine. This suggests that much of the rise of pNGAL could be contributed by decreased clearance due to the drop in filtration due to AKI.[9] Given that the universally available serum creatinine values from any of the time points were enough to eliminate any independent associations between pNGAL with long-term mortality, it brings into question the clinical utility of measuring pNGAL for the purposes of risk-stratification for long-term mortality after cardiac surgery. A number of urinary and blood biomarkers are being tested and marketed for diagnosis and risk-stratification of AKI. We recently demonstrated that five urinary biomarkers measured on post-operative days 1–3: NGAL (urinary), interleukin-18 (IL-18), kidney injury molecule-1 (KIM-1), liver fatty acid binding protein (L-FABP), and albumin were independent predictors of long term survival in patients with clinical AKI.[6] However, based on the results we present in this paper we can conclude that the value of pNGAL as an independent marker of long-term survival is weak.

Plasma and Urinary NGAL may represent distinct pathophysiology. Postoperative urinary NGAL is associated with long-term mortality (adjusted HR 2.52, 95% CI 1.86–3.42), whereas this study shows that postoperative plasma NGAL does not have a similar relationship.[6] Understanding the pathophysiology of NGAL production and excretion may offer some insight into this discrepancy. NGAL when associated with its ligand (the siderophores) and iron has been shown to promote growth and differentiation of the tubular structure of nephrons and may be protective in ischemic and nephrotoxic insults to the kidney.[15,16] NGAL is known to have distinct pools which regulate the way in which NGAL acts on target tissues which may explain differences in study outcomes when based on whether the NGAL pool tested is derived from the plasma or urine.[17] Plasma NGAL is produced mainly in the liver and white blood cells, freely filtered by the glomerulus, and reabsorbed in the proximal tubule by a megalin-mediated uptake.[18] The elevation of plasma NGAL in detection of AKI may be a result of poor clearance of extra-renal pool of NGAL. We demonstrated previously that the prognostic signals provided by pNGAL both for AKI[5] and long-term mortality (this study) are markedly attenuated when adjusted for change in serum creatinine. In contrast, urinary NGAL levels are not influenced by decreased clearance due to decrements in eGFR. For example, in a cohort of CKD patients, pNGAL levels were much more correlated with eGFR (R^2^ = -0.53, p < 10^–5^) than urinary NGAL levels (R^2^ = 0.14, p < 10^–5^).[11] Urinary NGAL concentration reflects the sum total of decreased reabsorption of filtered NGAL and local NGAL synthesis in the thick ascending limb of Henle and collecting ducts. Point of care or rapid NGAL tests for pNGAL are being sold and are currently available as a tool for early diagnosis of AKI in several countries outside the United States.[19–21] However, we feel that there needs to be a greater distinction between these two pools of NGAL as they clearly signify discrete pathologies before this becomes part of clinical practice. This study highlights the importance of considering plasma NGAL as a clearly distinct pool than urinary NGAL.

There is some evidence establishing association between baseline pNGAL value and long-term mortality (in the absence of AKI). A study of community-dwelling older adults demonstrated that a baseline NGAL value of > 192 ng/ml was independently associated (even after adjusting for eGFR) with cardiovascular disease outcomes and mortality after 11 years of follow up.[22] In a cohort of 5599 community dwelling adults, baseline pNGAL was independently associated with cardiovascular mortality at 10-year follow-up.[23] Similarly, in our study pre-operative (baseline) pNGAL was associated with increased mortality if it was detectable (lower limit of detection 60 ng/ml), even after adjustment for eGFR.

Our study has several limitations. First, our study lacks data on cause of death, occurrence of CKD or CKD progression or ESRD on long term follow-up. Second, as mentioned above, follow up time of 3 years may be insufficient to predict long term survival, however, this is the longest follow up thus far for any pNGAL study in the setting of AKI. Third, we did not adjust the pNGAL values for the white blood cell count; however this adjustment may, in fact, further hamper the predictive ability of pNGAL to mortality. Finally, the decision to analyze pNGAL as tertiles was post-hoc and may be a source of bias. However, until there is an established and validated cut-off for this test for prediction of long-term mortality, its analyses can only be performed by dividing it into quantiles. This study does have several strengths. It is the largest study to date of kidney biomarkers with assessment of long-term outcomes. We had sufficient number of deaths to allow us to adjust for several important pre- and intra-operative covariates. Multiple clinical centers participated, the processes used to collect and store the biospecimens were standardized across sites, and we used validated assays for all biomarker measurements.

In conclusion, our study shows that pre-operative pNGAL levels were independently associated with 3-year mortality after cardiac surgery. While post-operative pNGAL levels were also associated with 3-year mortality, this relationship was not independent of changes in serum creatinine. These findings suggest that while pre-operative pNGAL adds prognostic value for mortality beyond routinely available serum creatinine, post-operative pNGAL measurements may not be as useful for this purpose. Since serum creatinine values are universally available, it brings into question the clinical utility and cost-effectiveness of measuring plasma NGAL for long-term mortality risk-stratification. Future studies that examine pNGAL should account for changes in filtration for full independence assessment.

## Supporting Information

S1 Table Baseline Characteristics by Pre-op Plasma NGAL Tertiles. Abbreviations and definitions: Data presented as number (percent) unless otherwise specified. LVEF- left ventricular ejection fraction, eGFR- estimate glomerular filtration rate, ICU- intensive care unit, LOS- length of stay, IQR- interquartile range; Clinical AKI defined as change in serum creatinine of ≥ 100% from pre- to the peak post-operative value. Oliguria defined as a patient who had <125 cc in 6 hours or <500 cc urine output in 24 hours; Note: To convert serum creatinine values to mmol/L, multiply by 88.4.
(PDF)

S2 Table Correlation (R^2^) between Plasma NGAL and other variables. (PDF)

S3 Table Associations with Mortality separately by AKI and no AKI. *Mortality rate per 1000 patient-years adjusted for site. §Model 1: Adjusted for Age (per year), sex, white race, CPB time > 120 minutes, non-elective surgery, pre-op eGFR, diabetes, hypertension, centre, Congestive heart failure (CHF), Myocardial Infarction (MI), Pre-op urine albumin to creatinine ratio, and Type of surgery (CABG or valve vs. all others).
(PDF)
