# Supplementary material for: Association of Perioperative Plasma Neutrophil Gelatinase-Associated Lipocalin Levels with 3-Year Mortality after Cardiac Surgery: A Prospective Observational Cohort Study
Source: PLoS One. 2015 Jun 8;10(6):e0129619. doi: 10.1371/journal.pone.0129619 (PMC4460181; doi:10.1371/journal.pone.0129619)
Supplement: S1 Table — Abbreviations and definitions: Data presented as number (percent) unless otherwise specified. LVEF- left ventricular ejection fraction, eGFR- estimate glomerular filtration rate, ICU- intensive care unit, LOS- length of stay, IQR- interquartile range; Clinical AKI defined as change in serum creatinine of ≥ 100% from pre- to the peak post-operative value. Oliguria defined as a patient who had <125 cc in 6 hours or <500 cc urine output in 24 hours; Note: To convert serum creatinine values to mmol/L, multiply by 88.4. (PDF) [file pone.0129619.s001.pdf]

Supplementary Table 1. Baseline Characteristics by Pre-op Plasma NGAL Tertiles

	Pre-op Plasma NGAL Tertiles			
	**T1: <60** N=589	**T2: 60-77** N= 156	**T3: 77-1300** N=376	**P value**
**Demographics**				
Age at the time of surgery, mean (SD)	70.93 (10.07)	71.84 (9.65)	72.49 (10.05)	0.04
Men	395 (66%)	103 (64%)	280 (73%)	<0.001
White Race	551 (93%)	146 (93%)	356 (94%)	0.72
**Medical History (time of surgery)**				
Diabetes	229 (39%)	63 (40%)	147 (39%)	0.95
Hypertension	460 (78%)	119 (76%)	308 (82%)	0.22
LVEF <40%	60 (10%)	8 (5%)	44 (12%)	0
Previous myocardial infaction	151 (26%)	42 (27%)	96 (25%)	0.95
eGFR (mL/min per 1.73 m^2), mean (SD)	71.92 (17.87)	68.4 (17.03)	60.17 (20.38)	<.001
Pre-op eGFR stage 1	98 (16%)	17 (11%)	24 (6%)	<.001
Pre-op eGFR stage 2	354 (59%)	90 (56%)	169 (44%)	
Pre-op eGFR stage 3	143 (24%)	50 (31%)	163 (43%)	
Pre-op eGFR stage 4	7 (1%)	3 (2%)	25 (7%)	
Serum Creatinine (mg/DL), median [IQR[	1 (0.8, 1.1)	1 (0.9, 1.2)	1.1 (1, 1.4)	<.001
Urine albumin to creatinine				
<=10.0	233 (39%)	68 (43%)	103 (27%)	<0.001
10.0-30	186 (31%)	41 (26%)	113 (30%)	
30-300	143 (24%)	39 (25%)	131 (35%)	
>=300	29 (5%)	9 (6%)	30 (8%)	
**Surgical Characteristics**				
Elective Surgery	467 (79%)	121 (77%)	309 (82%)	0.36
Surgery,				
CABG	310 (51%)	85 (53%)	156 (41%)	0.01
CABG + Valve	130 (22%)	34 (21%)	96 (25%)	
Valve	162 (27%)	41 (26%)	129 (34%)	
Off-pump	48 (8%)	23 (15%)	41 (11%)	0.08
Re-do surgery	9 (2%)	2 (1%)	8 (2%)	0.72
Perfusion time (minutes), mean (SD)	112.64 (55.67)	109.21 (67.1)	119.43 (63.98)	0.08

Cross-clamp time (minutes), mean (SD)	75.15 (41.5)	73.13 (47.09)	83.86 (47.66)	0.006
**Post-operative Complications**				
Clinical AKI	20 (3%)	3 (2%)	25 (7%)	0.02
Oliguria in first day	6 (1%)	3 (2%)	6 (2%)	0.59
Delta Peak Serum Creatinine (mg/dL), mean (SD)	0.17 (0.33)	0.2 (0.29)	0.27 (0.47)	<.0001
ICU LOS, median [IQR]	2 [1, 3]	2 [1, 3]	2 [1, 4]	<0.001
Hospital LOS, median [IQR]	6 [5, 8]	6 [5, 9]	7 [5, 9]	<0.001

Abbreviations and definitions:
Data presented as number (percent) unless otherwise specified.
LVEF- left ventricular ejection fraction, eGFR- estimate glomerular filtration rate, ICU- intensive care unit, LOS- length of stay, IQR- interquartile range; Clinical AKI defined as change in serum creatinine of ≥ 100% from pre- to the peak post-operative value. Oliguria defined as a patient who had <125 cc in 6 hours or <500 cc urine output in 24 hours;
Note: To convert serum creatinine values to mmol/L, multiply by 88.4.
