# Supplementary material for: Association of Perioperative Plasma Neutrophil Gelatinase-Associated Lipocalin Levels with 3-Year Mortality after Cardiac Surgery: A Prospective Observational Cohort Study
Source: PLoS One. 2015 Jun 8;10(6):e0129619. doi: 10.1371/journal.pone.0129619 (PMC4460181; doi:10.1371/journal.pone.0129619)
Supplement: S2 Table — (PDF) [file pone.0129619.s002.pdf]

S2 Table. Correlation (R^2) between Plasma NGAL and other variables

Plasma NGAL			
**Variable**	**Pre-op**	**0-6 hour**	**Peak**
**Serum Creatinine**	0.298	0.347	0.381
**CKD-EPI GFR**	-0.272	-0.369	-0.376
**Delta S. Cr**	N/A	0.241	0.283
**Blood Cystatin C**	0.33	0.34	0.361
**Urine NGAL**	0.084	0.361	0.296
**Pre-op Plasma NGAL**	N/A	0.326	0.342
