# Supplementary material for: Association of Perioperative Plasma Neutrophil Gelatinase-Associated Lipocalin Levels with 3-Year Mortality after Cardiac Surgery: A Prospective Observational Cohort Study
Source: PLoS One. 2015 Jun 8;10(6):e0129619. doi: 10.1371/journal.pone.0129619 (PMC4460181; doi:10.1371/journal.pone.0129619)
Supplement: S3 Table — *Mortality rate per 1000 patient-years adjusted for site. §Model 1: Adjusted for Age (per year), sex, white race, CPB time > 120 minutes, non-elective surgery, pre-op eGFR, diabetes, hypertension, centre, Congestive heart failure (CHF), Myocardial Infarction (MI), Pre-op urine albumin to creatinine ratio, and Type of surgery (CABG or valve vs. all others). (PDF) [file pone.0129619.s003.pdf]

S3 Table. Associations with Mortality separately by AKI and no AKI

	No AKI		AKI		Interaction p-value
	**Mortality rate ***	**HR Model 1§**	**Mortality rate***	**HR Model 1§**	
**Plasma NGAL first post-op**					
T1	36.8	1.0 (referent)	70.4	1.0 (referent)	
T2	42.7	0.82 (0.47, 1.44)	81.7	1.48 (0.71, 3.11)	0.41
T3	40.5	0.73 (0.38, 1.39)	77.4	1.31 (0.65, 2.67)	0.04
**Plasma NGAL Peak**					
T1	31.0	1.0 (referent)	58.0	1.0 (referent)	
T2	47.1	1.21 (0.69, 2.12)	88.1	1.31 (0.61, 2.83)	0.84
T3	47.2	1.06 (0.56, 2.02)	88.3	1.37 (0.66, 2.84)	0.29

*Mortality rate per 1000 patient-years adjusted for site

§Model 1: Adjusted for Age (per year), sex, white race, CPB time > 120 minutes, non-elective surgery, pre-op eGFR, diabetes, hypertension, centre, Congestive heart failure (CHF), Myocardial Infarction (MI), Pre-op urine albumin to creatinine ratio, and Type of surgery (CABG or valve vs. all others).
